# Socio-economic determinants in selecting childhood diarrhoea treatment options in Sub-Saharan Africa: A multilevel model

**DOI:** 10.1186/1824-7288-37-13

**Published:** 2011-03-23

**Authors:** Olatunde Aremu, Stephen Lawoko, Tahereh Moradi, Koustuv Dalal

**Affiliations:** 1 Department of Public Health Sciences, Karolinska Institutet, SE 17177 Stockholm, Sweden; 2 Southampton Health Technology Assessment Centre, School of Medicine University of Southampton, Southampton SO16 7PX. UK; 3 College of Medicine, University of Ibadan, Ibadan, Nigeria; 4 Departments of Medicine and Health Science, Centre for Health Technology Assessment Linkoping University, Sweden; 5 Division of Epidemiology, The institute of Environmental Medicine, Karolinska Institutet, Stockholm, Sweden

## Abstract

**Background:**

Diarrhoea disease which has been attributed to poverty constitutes a major cause of morbidity and mortality in children aged five and below in most low-and-middle income countries. This study sought to examine the contribution of individual and neighbourhood socio-economic characteristics to caregiver's treatment choices for managing childhood diarrhoea at household level in sub-Saharan Africa.

**Methods:**

Multilevel multinomial logistic regression analysis was applied to Demographic and Health Survey data conducted in 11 countries in sub-Saharan Africa. The unit of analysis were the 12,988 caregivers of children who were reported to have had diarrhoea two weeks prior to the survey period.

**Results:**

There were variability in selecting treatment options based on several socioeconomic characteristics. Multilevel-multinomial regression analysis indicated that higher level of education of both the caregiver and that of the partner, as well as caregivers occupation were associated with selection of medical centre, pharmacies and home care as compared to no treatment. In contrast, caregiver's partners' occupation was negatively associated with selection medical centre and home care for managing diarrhoea. In addition, a low-level of neighbourhood socio-economic disadvantage was significantly associated with selection of both medical centre and pharmacy stores and medicine vendors.

**Conclusion:**

In the light of the findings from this study, intervention aimed at improving on care seeking for managing diarrhoea episode and other childhood infectious disease should jointly consider the influence of both individual SEP and the level of economic development of the communities in which caregivers of these children resides.

## Introduction

Diarrhoea remains an important cause of morbidity and mortality among children aged five and below in most developing regions of the world. According to an estimate, diarrhoea is reported to be responsible for close to 2 million deaths annually in this age-group[1]. In sub-Saharan Africa (SSA), the occurrence of diarrhoea like other infectious disease has been associated with poverty [2-4]. Timely administration of oral rehydration salt (ORS), and more recently Zinc tablets have proved to be both more cost effective and efficacious as primary interventions for preventing diarrhoea morbidity [5-11]. Despite the availability of these interventions; there have been no decline to diarrhoea incidence as many children are not using the interventions[12]. As a result, diarrhoea disease continues to be a serious threat to children in many countries in SSA [13,14].

The socio-economic gradient of diarrhoea occurrence is well established in literature. However, it is important to know the extent to which it also determines where care is sought when managing diarrhoea. Although several studies have document the role of socioeconomic factors in care seeking for childhood diarrhoea [15-17], many of these studies have not been able to explore the role of socio-economic characteristics of the neighbourhood in shaping or hindering the treatment options by caregivers. There is evidence suggestive of major influence of socio-economic characteristics of neighbourhoods in access to, and utilization of care [18]. In particular, living in socio-economic deprived neighbourhoods has been well documented to be associated with less likelihood of seeking medical care[19]. Thus, examining various perspectives by which socio-economic factors can influence selection of treatment options for childhood diarrhoea is of high importance.

So therefore, to bridge the paucity of knowledge on the wider influencing role of socioeconomic status in childhood diarrhoea treatment, this study used multilevel modelling [20-22]. Multilevel modelling technique permits simultaneous investigation of the role that individual socio-economic position and neighbourhood socio-economic status has on selection of treatment options. Highlighting such would contribute to a greater understanding of what is needed to be done to mitigate less uptake of the recommended intervention.

## Methods

### Study participants and methods

The data analysed in this study was derived from the Demographic and Health Surveys (DHS)[23]conducted in eleven countries in sub-Saharan Africa (Benin, Burkina Faso, Cameroon, Ghana, Kenya, Liberia, Mali, Nigeria, Niger, Senegal, and Tanzania) between 2003 and 2008. The DHS surveys are a series of nationally representative household survey, that are normally conducted in most low and middle income countries by ICF Macro[23]. It employs a two-stage sampling procedure, with the first stage involving the selection of primary sampling units (PSUs); these are probability proportional to the size and represent the number of households within the PSU. The second stage uses a systematic technique to sample households from each of the selected PSUs units. The full details of the methods and procedure used in data collection in DHS surveys is provided elsewhere[23]. We pooled dataset from the selected countries, and analysed 12,988 data that were useable out of 14,964 care givers who had sought treatment for childhood diarrhoea two weeks prior to the survey.

#### Ethical considerations

This study is based on secondary analysis of existing survey data, with all personal identifying information removed. The survey instrument received ethical permission from the National Ethics Committee in the respective countries, and the institutional review board of ICF Macro Inc. The permission to use this data was by ICF Macro.

### Measures

The outcome measures for this study were the treatment options for childhood diarrhoea among the care givers of children aged five and below. The DHS interviewer specifically asked care givers in their respective local dialects "Were any of your children aged five and below passed loose watery stool with or without blood continuously for more than three times in any particular day in the last two weeks?" Caregivers were further probed about type of care that was sought. The type of facility the caregiver of a child with diarrhoea could report having sought treatment from was classified into: (1) medical centre which comprised of treatment received at any healthcare facility maintained by public, non-governmental or private entity; (2) Pharmacy or patient medicine vendors; (3) traditional healer (otherwise known as herbalist i.e. a local doctor who claimed to be a specialist in the use of herbs and other supernatural powers to treating ailments by appeasing deities using spiritual invocations); ( 4) homes or self treatment(this includes the use of ORS ) Briefly, ORS is a combination of salts and sugar mixed with safe water normally administered to replace lost fluid due to diarrhoea. This is usually prepared by mixing half a teaspoonful of salt with six teaspoonful of sugar in one litre of freshly boiled or ready to drink water and (5) no treatment.

The individual measures of SEP assessed in this study are as follows; caregiver's education (no education, primary, secondary &higher); caregiver's occupation (not working, manual, professional); partner's education (no education, primary, secondary &higher); partner's occupation (not working, manual, professional); and wealth index. The wealth index is the proxy indicator of the socioeconomic status, and was derived based on the scores allocated to each household possession. The total score was later divided into wealth quintiles: poorest, poor, middle, richer and richest. The principal component analysis (PCA) [24], was used to develop neighbourhood socioeconomic disadvantage index[25,26]. This index encompassed four variables which included proportion of caregivers; living in rural areas; who were unemployed; living below the poverty level (below 20% quintile), and who were uneducated. From this index, with a mean value of 0 and standard deviation of 1, neighbourhoods were classified into two categories (lower and higher) of socio-economic disadvantage. A higher score is indicative of most socio-economically disadvantaged neighbourhoods and lower score implies (least socio-economic disadvantaged neighbourhoods). Other covariates' such as age of caregivers were grouped as 15-24 years, 25-34 years, and 35 years and older, and place of residence as rural-urban respectively.

### Statistical analysis

The care givers' choice of treatment for childhood diarrhoea is a discrete choice, of which there are several other choice alternatives. The most commonly used discrete choice model is multinomial logit, within the random utility framework. This framework, assumes that an individual is rational and makes a choice based on preference which is governed by utility[27]. However, the hierarchical structure of our data where individual caregivers' are nested within households and communities warrants the use of multi-level multinomial modelling technique [20,28,29]. The full details of the statistical models employed in the analysis are provided in the Appendix. All analysis were performed using MLwiN 2.02 software[28].

## Results

### Descriptive statistics

The summary statistics of sample characteristics based on country, year of study, primary sampling units and treatment choice were presented in Table 1. The PSUs (clusters) varies from 345 in Niger to 888 in Nigeria. The sample size is between 526 in Ghana to 2,396 in Nigeria. Table 2.depicts the number and percentage of socioeconomic and demographic characteristics of the caregivers for the pooled sample tabulated over the five treatment choices. In all, a total of 12,988 caregivers of children with diarrhoea two weeks prior to the survey in 11 countries were analyzed in this study. Of these, 23% were urban dwellers and the remaining 77% lives in rural areas. Almost two-third (69%) of the caregiver's does not possess any formal education, most were in their middle aged 25-34 years (46%), and 30% were unemployed. More than half of the caregivers (52%) sought no treatment for childhood diarrhoea incidence. Out of the total sample, 21% sought treatment at medical centre, 9% patronized pharmacy stores and medicine vendors, 11% engaged in home care and only 6% received treatment from traditional healers or herbalist.

**Table 1:** Description of selected countries data sets by, primary sampling units, and sample size treatment choices

Country (Year of survey)	PSU	Sample size	No treatment	Medical centre	Pharmacy/ Vendors	Home care	Traditional
Benin (2006)	750	1,240	754	224	101	132	29
Burkina- Fasso (2003)	400	1,864	1,258	273	50	173	110
Cameron (2003)	466	1,072	592	149	129	129	73
Ghana (2007)	412	526	186	253	14	64	9
Kenya (2003)	782	782	534	169	44	0	35
Liberia (2007)	300	1055	188	496	185	95	91
Mali (2006)	408	1,416	892	157	38	198	131
Nigeria (2008)	888	2,396	811	724	549	229	83
Niger (2006)	345	1,616	939	277	65	185	150
Senegal (2006)	377	2,060	1183	341	95	293	148
Tanzania (2006)	475	937	360	291	98	165	23

Source: Demographic and Health Surveys of the selected countries

PSU: Primary sampling units

**Table 2:** Socio-demographic profiles of caregivers according to treatment options

Variables	Sample size n (%)	No treatment n (%)	Medical centres n (%)	Pharmacy/ Vendors' n (%)	Home care n (%)	Traditional n (%)
Caregiver's education						
None	8,936 (68.8)	4,991 (55.9)	1,641 ( 18.4)	829 (9.3)	903 (10.1)	572 ( 6.4)
Primary	3,006 (23.1)	1,414 (47.0)	786 (26.8)	281 (9.6)	371 ( 12.3)	154 ( 5.1)
Secondary/Higher	1,046 (8.1)	404 (38.6)	363 (34.7)	77 ( 7.4)	161(15.4)	41 (3.9)
Partner's education						
None	7,941 (61.1)	4,582 (57.7)	1,345 (16.9)	702(8.8)	813(10.2)	499 (6.3)
Primary	2,866 (22.1)	1,386 (48.4)	696 (24.3)	285 (9.9)	348(12.1)	151 (5.2)
Secondary/Higher	2,181 (16.8)	841 (38.6)	749 (34.3)	200 (9.1)	274(12.6)	117 (5.4)
Caregiver's occupation						
Not working	3,860 (29.7)	2,102 (54.4)	791 (20.5)	347 (8.9)	414(10.7)	206 (5.3)
Manual	7,380 (56.8)	3,766 (51.0)	1,677 (22.7)	666 (9.0)	824(11.1)	447 (6.0)
Professional	1,748 (13.5)	947 (53.8)	322 (18.4)	174 (9.9)	197(11.3)	114 (6.5)
Partner's occupation						
Not working	668 (5.2)	273(40.8)	208 (31.1)	60 (8.9)	97(14.5)	30 (4.5)
Manual	8,587 (66.1)	4,461 (51.9)	1,879 (21.8)	835 (9.7)	914(10.6)	498 (5.8)
Professional	3,733 (28.7)	2,075(55.6)	703 (18.8)	292 (7.8)	424(11.4)	239 (6.4)
Caregiver's age						
15-24	3,948 (30.4)	2,118(53.6)	817 (20.7)	364 (9.2)	404 (10.2)	245 (6.2)
25-34	6,022 (46.4)	3,135(52.0)	1,325 (22.0)	538 (8.9)	684 (11.4)	340 (5.6)
35+	3,018 (23.2)	1,556 (51.6)	648 (21.5)	285 (9.4)	347 (11.5)	182 (6.0)
Place of residence						
Rural	9,989 (76.9)	5,425 (54.3)	1,973 (19.8)	901 (9.0)	1,054 (10.5)	636 (6.4)
Urban	2,999 (23.1)	1,384 (46.1)	817 (27.2)	286 (9.5)	371(12.4)	141 (4.3)
Household wealth						
Poorest	3,546 (27.3)	1,913 (53.9)	659 (18.6)	431 (12.1)	310 (8.7)	233 (6.6)
Poorer	3,209 (24.7)	1,690 (52.6)	621 (19.3)	338 (10.5)	337 (10.5)	223 (7.0)
Middle	2,747 (21.2)	1,436 (52.3)	595 (21.6)	203 (7.3)	348 (12.6)	165 (6.0)
Richer	1,986 (15.3)	1,028 (51.7)	496 (25.0)	141 (7.1)	239 (12.0)	82 (4.1)
Richest	1,500 (11.5)	742 (49.5)	419 (27.9)	74 (4.9)	201 (13.4)	64 (4.3)
Neighbourhood Socio- economic disadvantage						
Low	6,493 (49.9)	3,488 (53.7)	1,400 (21.6)	470 (7.2)	768 (11.8)	367 (5.6)
High	6,495 (50.0)	3,32 1(51.1)	1,390 (21.5)	717 (11.0)	667 (10.2)	400 (6.2)

### Multilevel multinomial regression analysis

Table 3.depicts the OR (odd ratio) from the multinomial multilevel models. The result shows that, relative to care givers with no education, those with primary education had 19% and 26% more likelihood of selecting medical centre and home care as against no treatments. Among those with higher education and relative to those with none; the likelihood of selecting medical centre and home care to no treatment were 47% and 72% higher respectively. A similar trend was observed with respect to the partner's education. Caregivers, whose partners had higher education compared to those whose partner had no formal education, has almost more than twice odds and twice likelihood of selecting medical centre and pharmacy to treat childhood diarrhoea than to no treatment. In addition, the likelihood of selecting home care was only 29% in those caregivers whose partner had higher education compared to those who had none. Whereas the result shows, that for care givers having partners with only primary education, relative to those who had none; the likelihoods of selecting medical centre, pharmacy and home care were 57%, 50%, 23% higher respectively, compared to no treatment.

**Table 3:** Multilevel-multinomial model estimates of treatment options by caregivers' socio- economic characteristics with no treatment as reference

Variables	Medical centres	Pharmacy/Vendors	Home care	Traditional
	
	OR 95% CI	OR 95% CI	OR 95% CI	OR 95%CI
*Fixed effects*				
Caregivers age				
35+ (ref)	1 -	1. -	1.	1 -
25-34	0.92 (0.82,1.02)	0.90 (0.75, 1.10)	(0.78 -1.06)	0.94(0.74,1.13)
15-24	0.86 (0.74,0.98)*	0.92 (0.74, 1.09)	0.80 (0.65,0.95)**	1.03(0.84,1.23)
Caregiver's education				
None (ref)	1 -	1 -	1 -	1 -
Primary	1.19 (1.08,1.30)*	1.01 (0.83,1.17)	1.26 (1.11,1.41)***	0.88 (0.61,1.10)
Secondary/Higher	1.47 (1.31,1.63)***	1.01 (0.92,1.29)	1.72 (1.50,1.94)***	0.76 (0.37,1.15)
Partner's education				
None (ref)	1 -	1 -	1	1 -
Primary	1.57 (1.46,1.68)***	1.50 (1.34,1.66)***	1.23 (1.08,1.38)**	1.10 (0.93,1.35)
Secondary/Higher	2.48 (2.35,2.61)***	2.00 (1.78,2.21)***	1.29 (1.10,1.48)**	1.60 (1.34,1.86)
Caregiver's occupation				
None (ref)	1 -	1 -	1	1.
Manual	1.16 (1.01,1.26)***	0.97(0.83,1.11)	1.12 (0.98,1.25)	1.18 (0.99,1.36)
Professional	0.98 (0.83,1.13)	1.28 (1.08,1.48) **	1.05 (0.87,1.26)	1.22 (0.97,1.47)
Partner's occupation				
None (ref)	1 -	1 -	1 -	1 -
Manual	1.08 (0.89,1.27)	1.04 (0.74,1.34)	0.84 (0.59,1.09)	0.99 (0.68,1.41)
Professional	0.77 (0.57,0.96)*	0.75 (0.44,1.06)	0.75(0.50,1.01)*	1.08 (0.66,1.50)
Household wealth				
Poorest(ref)	1 -	1 -	1 -	1 -
Poorer	1.04 (0.91,1.16)	0.86 (0.73,1.02)*	1.18 (1.00,1.33)*	1.07(0.87,1.27)
Middle	1.12 (0.99,1.25)	0.58 (0.39,0.77)***	1.14 (1.24, 1.58)***	0.93 (0.71,1.15)
Richer	1.10 (0.96,1.24)	0.45 (0.23,0.67)***	1.19 (1.00,1.39)	0.64 (0.37,0.91)
Richest	0.92 (0.74,1.10)	0.21(0.10,0.52) ***	1.16 ( 0.93,1.36)	0.68 (0.33,1.03)
Place of residence				
Urban (ref)	1-	1-	1-	1-
Rural	0.70 (0.86,1.10)***	0.44(0.26,0.62)***	0.82 (0.66,0.98)**	1.02(0.91,1.26)
Neighbourhoods socio-economic disadvantage		1-	1-	1-
High(ref)	1-	1.56(1.39,1.73)***	1.02 (0.88,1.16)	1.13(0.68,1.32)
Low	1.17(1.06,1.28)**			
*Random effects*				
Community multinomial Variance(SE)	1.17(0.03)***	1.50(0.06)**	1.31(0.04)***	1.49(0.08)***
*Intracluster correlation *(ICC) *(%)*	26.2	31.3	28.4	31.1

Abbreviations: OR, odds ratio; CI, confidence intervals; SE, standard error; ICC, intracluster correlation. *p < 0.05, **p < 0.01, ***p < 0.001

As shown in Table 3. Care givers who were manual workers and those that are professionals relative to those that were not working, were more likely to selecting medical centre and pharmacies to treat diarrhoea compared with no treatment. However, the likelihood of selecting medical centre and home care was much lowered for care givers whose partner is a professional relative to those whose partners had no occupation. The results shows further that, relative to their urban counterpart rural care givers had 30%, 56%, and 18% respectively less likelihood of selecting medical centre, pharmacy and home care compared to no treatment. Also, relative to those who are resident of highly socio-economic disadvantaged neighbourhood care givers who were resident of low socio-economic disadvantaged neighbourhoods were 17% and 56% more likely to select medical centre and pharmacy compared to no treatment. As stated in the random effect part of Table 3, it was revealed that almost 26%,28%, 31%, 31% variability in the log likelihoods of caregivers decision to select medical centre, home care, pharmacy or traditional healers compared to no treatment could be attributed to other unobserved characteristics at the community-level(r*= *1.17; *p *< .0001, r*= *1.31; *p *< .0001,r*= *1.50; *p *< .002, and r*= *1.49; *p *< .0001)., respectively. The effect remained significant, even after controlling for various individual and community level variables.

In relation to the household wealth index, relative to the poorest caregiver, the care giver at the middle quintile of wealth index and the poorer (20% above the poverty line) were more likely to select home care to manage childhood diarrhoea than to no treatment. Whereas, care givers from the richest households had 79% less likelihood of selecting pharmacies to treat diarrhoea relative to those from the poorest household.

## Discussion

The central aim of this study is to investigate the association between socio-economic status and selection of treatment for childhood diarrhoea among care givers in sub-Saharan Africa using multilevel multinomial regression analysis. The result shows that, caregiver's choice of treatment for childhood diarrhoea depends on several individual and neighbourhood measures of socio-economic status. Specifically, at the individual level, the analysis indicate that choice of medical centre for managing childhood diarrhoea was highly associated with the caregiver's and her partner's educational attainments. Highly educated caregivers had a higher odd of utilizing medical centre for managing childhood diarrhoea; this finding is in contrast to a study conducted in another developing region of the world with high prevalence of childhood diarrhoea [30]. Whereas, our findings are compatible with those of many others [31-33], that have documented positive association between maternal education and choice of medical centre for managing childhood diarrhoea. The finding that partners education is associated with choice of medical centre is in consonance with what had been reported earlier [33,34].This finding, further confirms the protective role of fathers education[35], as an additional reinforcement factor for mothers decision to seek appropriate care when managing childhood illness. The positive association between parental education and choice of medical centre as noted in this study further reverberates its importance for child survival in developing world [36].

In sub-Saharan Africa, fathers are the overall head of the household and sometimes decides where care is sought [37]. Hence, it is not surprising as noted in this study that, caregiver's partner's education, is associated with patronage of pharmacy store and medicine vendors and to some extent home care for managing diarrhoea episode. In addition, the decision of the educated caregivers to use home treatment, have been attributed to their ability to utilize health information wisely[38].

The influence of wealth status on caregiver's propensity to choose private and public medical centres when managing childhood illness has been documented in the literatures [30,39-41]. This is however, not noticed in this study. Although this study shows that, care givers from poorer households compared with those from poorest household engaged in home care as an alternative option for managing childhood diarrhoea. This finding is not surprising, it had been reported elsewhere that households sometimes do engaged in self medication especially when the cost of treatment in medical centre is high [42,43]. On the other hand this study joined other studies in documenting care givers' occupation, as another important factor influencing choice of treatment [33,39,41]. In this study, being a manual worker is closely associated with selection of medical centre, while being a professional working class is associated with patronage of pharmacy store or medicine vendors.

Geographic location, place of residence in particular, has been shown to be another form of disparity[44,45], which could prevent access to utilization of care[46]. This study shows that, residing in rural area, though statistically not significant, is associated with likelihood of patronizing traditional healers. Of the main interest in this study, is to examine the effect of neighbourhood socio-economic disadvantaged after controlling for individual SEP on clustering of selected treatment options around the neighbourhoods. The multilevel multinomial regression models indicate that, with all other factors being held constant, living in highly socio-economically disadvantaged neighbourhood is associated with less likelihood of using medical centre, pharmacy or vendors. The results of the between caregivers variation in the choice of treatment at the community-level indicates that several other factors which might be in part due to the caregivers neighbourhoods play a greater role in the individual choices. This finding suggests that, compositional characteristics of the caregivers are less important than that of the community with regards to individual choices.

### Study limitations and strengths

This study is without limitations and should be mentioned. First, the findings from this study are based on data from cross-sectional survey and the initiation of the caregivers to the health system for managing childhood diarrhoea. It is however, possible for care to be sought from more than one provider upon the failure of the initial treatment. Second, we used an indirect measure of household wealth status. However, as DHS surveys do not collect data on income, the use of household possession has been shown to be relevant in developing country settings[47]. Finally, the analysis was based on self reported diarrhoea morbidity has reported by the care giver which could be subject to recall bias. In spite of these limitations, the strength of our study lies in the unique characteristics of the DHS. The DHS are nationally representative, and allows for findings to be generalized across the entire country. In addition, the design and the variables included in the survey are the same across countries, and thus, making it possible for us to be able to pool the data from these countries.

## Conclusions

In sum, this study has revealed that regardless of where individual care giver resides, treatment choices would be similar based on their level of educational attainments (compositional effects). On the other hand, the characteristics of the neighbourhoods level of economic development, accounts for variation from caregiver to caregiver in their choice of treatment at the community level (contextual effects). Hence, interventions aimed at improving appropriate care seeking for managing childhood diarrhoea must take into consideration care giver's SEP and the level of socio-economic development of the community in which each care giver resides.

## Abbreviations

ORS: Oral rehydration salts; DHS: Demographic and Health Surveys; OR: Odd Ratio; SSA: Sub-Saharan Africa; PSUs: Primary sampling units; SEP: Socio-economic position; PCA: Principal component analysis

## Competing interests

The authors declare that they have no competing interests.

## Authors' contributions

OA, SL, KD and TM were involved in the conception of the study. OA carried out data extraction. OA conducted statistical analysis and was checked for correctness by SL and KD. OA drafted the paper with contributions from the co-authors. All authors read and approved the final manuscript prior to submission.

## Appendix A. Statistical method

In this analysis, no treatment was used as the reference category and a set of *t- *1 logistic regressions were computed for the four remaining treatment options. These options are then, contrasted one after the other against no treatment (reference). Thus we specified a multilevel multinomial regression model as follows:(1)

Where *S *take values from 1 to t- 1, the subscript *s *denotes a separate set of intercepts for the reference and the four remaining set of categories. The caption ***β***_***0j***_^***(s) ***^depict the fixed part of the model and was interpreted as the effect of a 1-unit increase in ***X ***(that the set of predators variables (socio-economic variables) on the log odds of selecting category ***s ***(i.e medical centre or any other categories) other than the reference category *t *(no treatment). The terms in the brackets in the equation represents the random effects associated with the primary sampling units at the community level. These are assumed to be normally distributed with a mean value of 0 and different variances. The random effects are measures of variations, and are contrast specific as indicated by the subscript *s*, since different unobserved factors at each level may affect each contrast. For this analysis, the intra-clusters correlation (ICC) was used as a measure of random effects [48]. We based the regression and variance parameters on penalized quasi-likelihood (PQL) estimation, using second order Taylor series linearization.
